# Patterns in Duration of Emergency Department Boarding and Variation by Sociodemographic Factors

**DOI:** 10.5811/westjem.42477

**Published:** 2025-11-26

**Authors:** Christiana K. Prucnal, Melissa A. Meeker, Martin Copenhaver, Paul S. Jansson, Rebecca E. Cash, William Hillmann, Steven Knuesel, Wendy Macias-Konstantopoulos, Jonathan D. Sonis

**Affiliations:** * Harvard Medical School, Boston, Massachusetts; † Massachusetts General Hospital, Department of Emergency Medicine, Boston, Massachusetts; ‡ Brigham and Women’s Hospital, Department of Emergency Medicine, Boston, Massachusetts; § Johns Hopkins University School of Medicine, Department of Emergency Medicine, Baltimore, Maryland; || Massachusetts General Hospital, Department of Medicine, Boston, Massachusetts; # Newton Wellesley Hospital, Newton, Massachusetts

## Abstract

**Introduction:**

Emergency department (ED) boarding negatively affects patient outcomes, increasing length of stay, hallway care, and mortality. Prior research found disparities in capacity metrics like hallway care based on patient race and ethnicity. However, whether boarding differs by demographics is not well characterized. We examined boarding variation by sociodemographic factors in a hospital with a standardized bed-prioritization process. We hypothesized that a structured inpatient assignment method may be associated with reduced boarding inequity.

**Methods:**

This single-center, retrospective, cohort study included adult patients boarding in the ED after admission to the non-intensive care inpatient medicine service between February 2020–February 2023 at an urban, academic, tertiary-care hospital with > 110,000 annual ED visits. Primary outcome was time from admission order to inpatient bed transport. Patient demographics (age, sex, race/ethnicity, language, insurance, and housing status), visit characteristics (Emergency Severity Index, time, and day), and bed request features (telemetry, sitter need, and isolation precaution) were obtained via the medical record. We assessed for bivariate relationships between boarding time and demographics with descriptive statistics and analysis of variance using adjusted and unadjusted regression analyses with generalized estimating equations to account for patient-level correlation.

**Results:**

In total, 22,291 encounters were included. Average age was 64 (SD ±19) years, and 47% were female. Approximately 12% identified as Hispanic, 70% as non-Hispanic White, and 10% as non-Hispanic Black. Most (97%) boarded ≥ 120 minutes. In adjusted analyses, patients with Medicaid waited an additional 85 minutes (95% CI 49–121), and patients with Medicare waited an additional 67 minutes (95% CI 32–103) compared to those with commercial coverage (both P < .001, respectively). Non-Hispanic Black patients boarded 14 minutes longer (95% CI 22–51), and non-English primary language speakers boarded 15 minutes longer (95% CI 17–47) than non-Hispanic White patients and English primary language speakers, respectively, although these two findings were not statistically significant.

**Conclusion:**

Among adult patients admitted to the inpatient medicine service, non-commercial insurance such as Medicaid and Medicare was significantly associated with longer ED boarding, whereas race/ethnicity and primary language were not. Further study should determine whether these findings are replicated elsewhere, how this impacts patients, and whether targeted intervention can reduce inequities.

## INTRODUCTION

Emergency department (ED) boarding, which describes admitted patients receiving care in the ED while awaiting inpatient bed availability, increasingly strains healthcare capacity.^1^–^3^ Its effects are felt by ED patients, hospital staff, and admitted patients alike, and the issue is steadily worsening.^4^–^6^ Multiple studies demonstrate that ED boarding negatively affects patient outcomes.^1^–^3^,^7^ Boarding has been linked to an increase in ED length of stay (LOS), hallway care, poor patient satisfaction, longer hospital stays, preventable disability, and higher in-hospital mortality.^3^,^8^–^10^ It contributes to greater numbers of patients leaving the ED prior to completion of their evaluation and is tied to an increase in medical errors as well as malpractice action.^11^–^13^ In the current environment of hospital crowding, ED boarding is often directly correlated with hallway care, leading to decreased patient privacy and at times inadequate history and physical exam.^14^ Healthcare worker burnout is also associated with boarding, perpetuating the mismatch between inadequate healthcare resources and unmet patient need.^5^,^15^ To an extent, the supply/demand imbalance in hospital resources that drives ED crowding follows established temporal patterns. Weekends are often down-staffed and can be less busy and with reduced acuity than weekdays, and certain days of the week or periods of the day (ie, late morning and afternoon hours) are often the most crowded.^16^–^18^

Distinct from crowding and boarding, treatment disparities based on non-clinical patient characteristics are well-described in the ED setting. Triage assessments have been found to vary by patient race and sex.^19^–^22^ Pain medication administration has been shown to be inequitable based on patient race and ethnicity.^23^,^24^ Non-White, Spanish-speaking, or Medicaid-covered patients are more likely to be queue-jumped and roomed to a hallway spot.^25^ Several studies have found disparities in the use of hallway care based on patient race and ethnicity. 26,27 This aligns with findings from a related body of literature on demographic-based disparities in acute care and outpatient settings seen across lines of race and ethnicity, sex, age, and insurance status.^28^–^33^ Among specific findings are race-based disparities in door-to-needle time, heart-failure cardiology care, and thrombolysis metrics among patients with ischemic stroke, sex-based gaps in acute ST-segment elevation myocardial infarction outcomes, gender discrepancies in acute pulmonary embolism testing and treatment, age-based discrimination in clinical trials and organ transplant eligibility, and increased gatekeeping behaviors and barriers to healthcare access experienced by patients with Medicaid as compared to those with commercial insurance.^28^–^34^

However, current data are limited and conflicting on whether ED boarding varies by patient sociodemographic characteristics. One study found that men boarded longer than women and that those ≥ 75 years of age boarded for less time than their younger counterparts, while another study corroborated this gender disparity but found prolonged boarding time was associated with older age.^35^,^36^ To our knowledge at the time of this writing, only two studies have examined racial disparities in ED wait times for inpatient beds. One single-site study found that Black patients with high-acuity presentations boarded significantly longer than White patients, and the same racial disparity was seen with those presenting for psychiatric admission.^36^ Another multisite analysis similarly found both non-intensive care unit (ICU) admissions and ICU admissions had longer waiting periods for Black patients than non-Black patients, although the study looked at among-hospital rather than within-hospital differences on adjusted analyses, and the authors were unable to include data on bed-prioritization processes or distinguish ED LOS from boarding.^37^ Further study is needed to determine whether boarding patterns vary significantly based on these characteristics.

**Population Health Research Capsule**

What do we already know about this issue?

*Studies show healthcare disparities across race, sex, age, and insurance. Emergency department (ED) boarding negatively affects outcomes, but whether it differs by demographics is not well characterized*.

What was the research question?

*In a hospital with a standardized bed-prioritization process, does ED boarding vary significantly by sociodemographic factors?*

What was the major finding of the study?

*Publicly insured patients boarded significantly longer: Medicaid +85 minutes (95% CI 49–121; P < .001); and Medicare +67 minutes (95% CI 32–103; P < .001), compared to private insurance*.

How does this improve population health?

*ED boarding disproportionately affects socioeconomically vulnerable populations, highlighting a need to investigate possible contributing and mitigating factors*.

The criteria used by hospitals to determine when an admitted patient moves from the ED to the inpatient unit are not homogenous, and protocols impacting patient throughput and, thus, ED boarding duration differ by institution. Bed-assignment protocols vary considerably from those driven directly by individual staff decision-making where a flow manager works with the inpatient team to prioritize ED patients for the next available inpatient bed, to those that are standardized or assisted by decision-technology using objective criteria such as patient age, sex, and isolation requirements.^38^,^39^ In a hospital setting where patient throughput from ED to ready inpatient bed is largely based on a transparent algorithm considering objective, standardized criteria, we examined whether duration of ED boarding varied significantly based on patient sociodemographic factors.

## METHODS

### Study Design

This single-center, cohort analysis used retrospective chart review. The institutional review board (IRB) determined this protocol was exempt from informed consent (protocol #2023P000707).

### Study Setting and Population

The study included consecutive adult patients who boarded in the ED prior to transport to their inpatient bed after admission to the non-intensive care medicine service between January 1, 2022–December 31, 2023, at an urban, academic, tertiary hospital with approximately 110,000 annual ED visits. We focused on general medicine admissions rather than specialty service admissions such as to cardiology or oncology because admissions to other services at this institution require approval by a specialist from that service, a process we considered less generalizable to outside institutions. This hospital has approximately 1,000 beds and nearly 45,000 admissions annually.^40^ At the site of this study, patient throughput from ED to ready inpatient bed is guided by an algorithm prioritizing time since bed request and matching bed availability.

Matching bed availability is according to transparent objective criteria including the floor’s level of care, the floor’s service (eg, medical or surgical), the patient’s self-identified gender and patient’s precautions (for semi-private rooms), non-cohortable infection (requires private room), and special room type (eg, negative pressure, positive pressure, lead-lined). This process is overseen by specially trained admitting staff who follow these principles. Although chiefly based on these standardized factors, opportunity for clinical judgment and discretion does remain primarily at two junctures. First, the ED resource nurse or a healthcare professional in a similar role may add priority based on a clinical need (such as for a patient with dementia and high delirium risk, or a patient with high risk for skin breakdown). Second, the clinician signing the admission order may select the need for a “Level 1” geographically-based floor team or a “Level 2” non-localized floor team, the latter of which is associated with shorter wait times due to greater supply. Level 2 beds are reserved for admissions with reduced complexity and lower monitoring frequency needs, and although guiding criteria exist for this selection between Level 1 or 2, clinician discretion remains.

Operationally at this institution, once an inpatient bed is ready and available for the admitted ED patient, two additional steps must be completed for the patient to physically be transported out of the ED. First, the ED clinical team must complete verbal pass-off to the inpatient team (separately by both clinician and nurse), after which they indicate with a button click in the electronic health record (EHR) (Epic Corporation, Verona, WI) that this pass-off has been completed. When the unit coordinator sees this indicated on their screen or is verbally notified that the pass-off is complete, they assign transport. We excluded from our investigation patients < 18 years of age, those requiring ICU admission, and interfacility transfers (which may be subject to multiple confounding effects including delays relating to imaging uploading).

### Data

We defined our primary outcome—boarding time—as the time from bed request order for admission to medicine to departure from the ED to that inpatient bed, and characterized it as a continuous variable. We obtained patient demographics (age, sex, race, ethnicity, language, insurance, and housing status), visit characteristics (Emergency Severity Index [ESI] as well as time and day), and bed request features (telemetry, sitter need, or infection control isolation precaution) via the EHR. Methods for medical record review followed standardized criterion including abstractor training, defined case-selection criteria, defined variables, use of abstraction forms, description of health record database, and IRB approval, as described by Worster et al.^41^

### Analysis

We performed a descriptive analysis of boarding time, patient demographics, visit characteristics, and bed-request features using mean (SD) or median (interquartile range [IQR]), depending on distribution for continuous variables, and *n* (proportion) for categorical variables. We assessed bivariate relationships between boarding time and patient demographics with descriptive statistics and ANOVA. We used both adjusted and unadjusted regression analyses with generalized estimating equations (GEE) to account for patient level correlation (Software R version 4.3.1); GEE is a method for modeling correlated data that produces a marginal model to generate estimates representative of the population average.^42^ We chose GEE over a Cox proportional hazard model (survival analysis) to produce estimates that allowed comparison in terms of length of time in minutes rather than hazard ratios. In the adjusted models, we controlled for visit characteristics and bed request features. Specifically, the confounding factors we adjusted for included ESI, time and day, need for telemetry, sitter, and infection control isolation precautions.

We examined race/ethnicity and primary language in separate models due to the highly correlated nature of these two variables and concern that introducing collinearity into the model would reduce our ability to detect the individual effect of each. As this is not a universal approach to addressing collinearity, we also included a GEE analysis with race/ethnicity and primary language included in the same model. We performed sensitivity analyses to evaluate the robustness of our results, including performing the regression analysis for the subgroup of patients who boarded ≥120 minutes, to align with our formal definition of a boarding patient and performing the regression analysis among the subgroup of patients with a single, bed request order. Multiple and changing bed request orders may indicate increased patient complexity or unmeasured work-flow issues that could confound findings, which prompted this second sensitivity analysis. An additional stratified analysis of insurance carrier by age group was performed post hoc.

## RESULTS

We included a total of 22,291 encounters in our analysis, with average patient age of 64 years (SD ±19), 47% of whom were female. Approximately 12% identified as Hispanic, 70% as non-Hispanic White, and 10% as non-Hispanic Black, with non-English primary language speakers comprising 15% of encounters. Most had Medicare coverage (56%), followed by Medicaid (25%), and commercial insurance (16%), and 5% had non-permanent housing status (Table 1).

Most boarding patients had a triage ESI of 2 or 3 (46% and 51%, respectively), and approximately 88% arrived to the ED during day or evening hours (7 am – 11 pm). Monday was the highest volume day in terms of ED arrivals for admitted patients (16%), followed by the remaining weekdays (15%), and weekend days (12%). Fewer than a quarter of bed requests had an active isolation order (23%), 9% required telemetry, and only 1% required a 1:1 staff observer (Supplemental Table 1).

Most bed request orders were placed during the evening shift (3 pm – 11 pm, 49%) and the highest proportion of requests were placed on Tuesdays with the lowest proportion of requests on Saturday and Sunday (16% vs 13% and 12%). Mean ED census at the time of bed request order was 178 patients (SD 27; median 178, IQR 39) and mean daily boarder census was 40 patients (SD 12; median 39, IQR 16). The vast majority (97% of patients) boarded 120 minutes, with a mean duration of 21 hours, and 35% boarded > 24 hours. (Supplemental Table 2)

Based on GEE analysis that included race/ethnicity in the model, we found variations in duration of ED boarding based on bed request features, departmental as well as temporal factors, and patient demographics. Special bed type, which was defined as requiring either telemetry or 1:1 observer staff, increased boarding time by 50 minutes (CI 95% 11–90; P = .01). Need for active isolation precaution (such as known methicillin resistant Staphylococcus aureus colonization, or positive COVID-19 infection status) also increased boarding duration by 112 minutes (95% CI 83–141; P < .001). Lower acuity patients who had received an ESI of 3–5 in triage, boarded significantly less time than higher acuity ESI 2 patients (131 minutes less for ESI 3, 364 minutes less for ESI 4–5; 95% CI 153–08; P < .001 and 95% CI −441, −286; P < .001, respectively), and highest acuity ESI 1 patients boarded 124 minutes longer than ESI 2 patients, although this comparison did not reach statistical significance (95% CI −27, 275; P = .11).

Multiple departmental factors were significantly associated with increased ED boarding duration. For every patient increase in boarder census, wait time for inpatient bed increased by 25 minutes (95% CI 23–26; P < .001). Day of the week significantly correlated with duration of boarding with longest waiting period occurring if admission order was placed on a Sunday, followed by Saturday, and shortest duration of boarding occurring if admission order was placed on a Thursday (248 additional minutes, P < .001; 168 additional minutes P < .001, and 212 fewer minutes P < .001, respectively, as compared with Monday admission wait times).

Patient demographics were also associated with how long the patient boarded in the ED. Non-Hispanic Black-identifying patients boarded 14 minutes longer (95% CI −22, 51; P = .45) and non-English primary language speakers boarded 15 minutes longer (95% CI −17, 47; P = .37) than Non-Hispanic White-identifying patients and English primary language speakers, respectively, although these findings were not statistically significant. Patients 65 years of age waited 29 minutes longer than those 18–64 years of age, although this did not remain statistically significant on adjusted analysis (95% CI 0–58; P = .05). Patients with non-commercial insurance waited significantly longer than patients with commercial coverage in adjusted models (those with Medicaid waited an additional 85 minutes and those with Medicare waited an additional 67 minutes; 95% CI 49, 121; P < .001, and 95% CI 32–103; P < .001, respectively). Patient sex and non-permanent housing status were not associated with a significant increase in ED boarding time (Table 2).

When adjusting for race/ethnicity and primary language in separate models due to the correlated nature of these two variables, results were not significantly different. Similarly, results did not significantly differ when we adjusted for race/ethnicity and primary language in the same model (Supplemental Table 3).

Sensitivity analyses examining patients who boarded ≥ 120 minutes and patients who had a single, bed request order did not substantially affect results (Supplemental Table 4). We expected collinearity between the two groups of patients 65 years of age and patients with Medicare coverage, but we also recognized that undetected collinearity may additionally be present between the 65 age group and those with Medicaid coverage. Given this, we stratified insurance carrier by age group and did not find a significant proportion of those 65 years of age insured by Medicaid. (Supplemental Table 5).

## DISCUSSION

We found differences in duration of ED boarding based on features of the bed request order, temporal and departmental factors, and patient demographics. The variation seen with the former elements, such as longer waits for an infection control isolation bed or admission requests made on a Monday, has rational underpinnings and has been previously described.^16^–^18^ However, the basis for disparities seen with insurance carrier is more challenging to explain. Recognizing that collinearity is present between 65 years of age and Medicare insurance, it is somewhat easier to imagine why those with Medicare had increased boarding time. One hypothesis is that the ED work-up for these often older, Medicare-insured patients is typically more extensive than that needed for the often younger, non-Medicare insured patients based on the increased comorbidities often accompanying advanced age.

At this institution, although admission order and inpatient-bed readiness are to some degree distinct and independent from completion of ED workup (ie, an inpatient bed may become ready while the patient still has ED tests in process), it is common practice for patients not to travel to their inpatient bed until tests that are potentially disposition-changing have resulted. This longer workup effect is likely compounded by a lead-time bias in decision to admit older Medicare patients, where the clarity that discharge is not advisable (and hence the bed request order) comes earlier in their ED stay as compared with younger, non-Medicare patients. On the other hand, the opportunity within this institution’s admission algorithm to introduce discretion in priority based on clinical factors (such as patient dementia and increased delirium risk), would more logically seem to mitigate this trend. These hypotheses would require further investigation to explore and confirm.

The consequences of increased ED boarding duration for the Medicare-insured older adult are perhaps even more important to consider than the root causes, as the population with advanced age is at highest risk for deleterious effects and iatrogenic harm from longer boarding, including increased mortality.^43^ Waiting in the ED often equates with conditions that are deliriogenic, can cause delays in home medication reconciliation, and permits circumstances where nursing-to-patient ratios are at times precarious.^3^ The increased likelihood of hallway bed placement, disruption of day/night circadian rhythms, and constant ambient noise in the ED are potent agents increasing risk for delirium, which itself is tied to increased mortality.^44^ Similarly, delays in medication reconciliation, which have been described in the setting of ED boarding, can contribute to significant morbidity and mortality.^45^

Why Medicaid insurance is associated with longer duration of ED boarding is unclear. Inherent in a designation as Medicaid-eligible is a scarceness in socioeconomic resources, and literature describes that this is the patient often unduly impacted by social determinants of health and marginalized from consistent and adequate healthcare.^25^,^29^ Considering the known harms associated with ED boarding, the finding that patients with Medicaid are disproportionately affected only furthers the substantial inequity. Additional investigation is needed to determine contributors to this finding, such as whether the Medicaid cohort over-indexes those with greater medical complexity, creating similar workup and admission decisions as those we hypothesize are experienced by the typically older Medicare population. As barriers to access primary and preventative care exist disproportionately for those with Medicaid, we must also consider whether the opposite pattern may be at play, whereby this cohort might instead be over-represented in admission for lower acuity conditions, which could be impacted by the discretionary elements of the bed prioritization process. This requires further study.

In contrast to prior investigations, we did not find a significant difference in duration of ED boarding based on patient race and, similarly, ethnicity and primary language did not correlate with longer boarding in our study.^36^,^37^ The reasons for this are unknown, but may be influenced by this hospital’s largely transparent, objective, and standardized algorithm for patient throughput from ED to inpatient bed. Future work should examine whether the design of the patient flow algorithm plays a significant role in ED boarding duration inequities.

It may seem surprising that patients with an ESI 1 or 2, indicating higher acuity on arrival, boarded for the longest duration in the ED. However, recalling that our inclusion criteria specify patients admitted to medicine, and not to an ICU, makes this less unexpected. These were likely patients of significant medical complexity and diagnostic uncertainty where ED boarding duration was reasonably longer. Furthermore, this captured a segment of patients who may have had their disposition and bed request order change multiple times during their ED stay (eg, from initial medicine admission request, to upgrade to ICU request, to eventual de-escalation to medicine admission again after the patient stabilized during their ED boarding period). In our institution, the ED boarding clock does not “reset” with each bed request change. Results of our sensitivity analysis adjusting for number of bed request orders did not suggest this as a significant source of confounding.

## LIMITATIONS

Our study had three main limitations. First, this was a single-site, retrospective investigation, and findings may not be generalizable to other settings including community, rural, or geographically distant hospitals. Second, categorization of race/ethnicity may have resulted in misidentification of multi-race/ethnicity individuals; although registration procedure is to ask and elicit a patient’s self-identified race/ethnicity, this practice is not always followed such as in the case of the unresponsive or non-verbal patient. Third, we did not adjust for chief complaint, indication for admission, or patient comorbidities; thus, it is possible that our capacity measures do not adequately capture confounding due to the COVID-19 epidemic, which occurred during the study period. It is possible that patients speaking a primary language other than English or those with non-commercial insrance may have had significantly differing distributions of diagnoses and comorbidities, which could have biased our results.

## CONCLUSION

Among adult patients admitted to medicine through the ED, we found significant differences in ED boarding times based on aspects of the bed request order, specific department-related and time-based factors, and patient demographics. Patients with non-commercial insurance boarded significantly longer in the ED before transfer to inpatient medicine beds as compared to patients with commercial insurance coverage. These differences are particularly concerning as the implications of increased boarding time likely pose the highest risk for harm to these potentially more vulnerable populations. It is possible that unmeasured confounders such as illness severity, structural bias, or deviations from protocol may have contributed to study results. Further work should investigate potential underlying administrative, clinical, and social factors behind these findings to determine whether intervention can effectively remediate these inequities.

## Supplementary Information



## Figures and Tables

**Table 1:** Demographics of adult emergency department patients admitted to the inpatient medicine service.

Demographics	Encounters (N = 22,291)
Age (years)	
mean (SD)	64 (19)
median (IQR)	66 (28)
Age (years), n (%)	
18–64	10,325 (46%)
> 65	11,966 (54%)
Sex, n (%)	
Female	10,450 (47%)
Male	11,841 (53%)
Race, n (%)	
White	16,179 (73%)
Black	2,461 (11%)
Asian	839 (4%)
American Indian or Alaska Native,	47 (0%)
Native Hawaiian or other Pacific Islander	18 (0%)
More than 1 race	138 (1%)
Other/unknown	2,609 (12%)
Ethnicity, n (%)	
Hispanic	2,593 (12%)
Non-Hispanic	18,771 (84%)
Unavailable	927 (4%)
Combined Race/Ethnicity, n (%)	
Hispanic	2,593 (12%)
Non-Hispanic White	15,559 (70%)
Non-Hispanic Black	2,288 (10%)
Other	1,851 (8%)
Primary Language, n (%)	
English	19,002 (85%)
Non-English	3,289 (15%)
Insurance, n (%)	
Commercial	3,653 (16%)
Medicaid	5,476 (25%)
Medicare	12,512 (56%)
Other	650 (3%)
Housing Status, n (%)	
Permanent	21,065 (95%)
Non-permanent	1,226 (5%)

*SD*, standard deviation; *IQR*, interquartile range.

**Table 2:** Multivariable generalized estimating equation analysis showing variation in emergency department boarding by encounter and patient characteristics.

Demographics		Estimate (minutes)	*P*-value	95% CI
Age (ref: 18–64)	>65	29	.05	[ 0, 58]
Sex (ref: Female)	Male	10	.38	[−12, 32]
Combined Race and Ethnicity (ref: Non-Hispanic White)	Hispanic	−7	.72	[−43, 30]
	Non-Hispanic Black	14	.45	[−22, 51]
	Other*	−7	.74	[−50, 35]
Insurance (ref: Commercial)	Medicaid	85	< .001	[ 49, 121]
	Medicare	67	< .001	[ 32, 103]
	Other	97	.01	[ 21, 172]
Housing (ref: Permanent)	Non-Permanent	25	.35	[−27, 76]
Special Bed Type (ref: No)	Yes	50	.01	[ 11, 90]
Bed Precaution (ref: No)	Yes	17	.81	[−20, 155]
Time of Bed Request (ref: 7 AM – 3 PM)	3 PM–11 PM	232	<0.001	[ 204, 260]
	11 PM – 7 AM	177	<0.001	[ 145, 209]
Bed Request Day of Week (ref: Monday)	Tuesday	−117	<0.001	[−156, −78]
	Wednesday	−160	<0.001	[−199, −22]
	Thursday	−212	<0.001	[−251, −174]
	Friday	−155	<0.001	[−197, −114]
	Saturday	168	<0.001	[ 122, 214]
	Sunday	248	<0.001	[ 202, 294]
Active Isolation Precaution (ref: No)	Yes	112	<0.001	[ 83, 141]
Acuity per Emergency Severity Index (ESI) (ref: ESI of 2)	ESI 1	124	0.11	[−27, 275]
	ESI 3	−131	<0.001	[−153, −108]
	ESI 4–5	−364	<0.001	[−441, −286]
	ESI unavailable	−145	0.03	[−278, −11]
Emergency Department Census**		1	0.003	[ 0, 2]
Boarder Census		25	<0.001	[ 23, 26]

* Other insurance = Veterans Affairs, self-pay, Workers Compensation.

** For each additional patient increase in ED census, boarding time increased by one minute.

Note: Refer to Supplemental Table 3 for results of model that includes primary language.
