# Supplementary material for: Patterns in Duration of Emergency Department Boarding and Variation by Sociodemographic Factors
Source: West J Emerg Med. 2025 Nov 26;26(6):1640–7. doi: 10.5811/westjem.42477 (PMC12698171; doi:10.5811/westjem.42477)
Supplement: Supplementary file 1 [file wjem-26-1640-s001.pdf]

Supplemental Table 1. Patient Admission Characteristics

Arrival Time, n (%)		
	7a-3p	10,221 (46%)
	3p-11p	9,261 (42%)
	11p-7a	2,809 (13%)
Arrival Day of Week, n (%)		
	Monday	3,590 (16%)
	Tuesday	3,362 (15%)
	Wednesday	3,270 (15%)
	Thursday	3,301 (15%)
	Friday	3,411 (15%)
	Saturday	2,722 (12%)
	Sunday	2,635 (12%)
Emergency Severity Index (ESI), n (%)		
	1	139 (1%)
	2	10,230 (46%)
	3	11,385 (51%)
	4-5	350 (2%)
	Missing	187 (1%)
Telemetry; Monitor		
	No	20,211 (91%)
	Yes	2,080 (9%)
Sitter; Suicide Precautions		
	No	22,138 (99%)
	Yes	153 (1%)
Active Isolation Order, n (%)		
	No	17,160 (77%)
	Yes	5,131 (23%)

Supplemental Table 2. Departmental and temporal admission characteristics.

Bed request time, n (%)	7a-3p	6,253 (28%)
	3p-11p	10,971 (49%)
	11p-7a	5,067 (23%)
Bed request day of week, n (%)	Monday	3,293 (15%)
	Tuesday	3,481 (16%)
	Wednesday	3,305 (15%)
	Thursday	3,269 (15%)
	Friday	3,314 (15%)
	Saturday	2,971 (13%)
	Sunday	2,658 (12%)
Emergency department census	mean (SD)	178 (27)
	median (IQR)	178 (39)
Boarder census	mean (SD)	40 (12)
	median (IQR)	39 (16)
Boarding time (hours)	mean (SD)	21 (15)
	median (IQR)	19 (19)
Boarding time (hours), n (%)	<2	719 (3%)
	2 to <6	3,330 (15%)
	6 to <12	3,117 (14%)
	12 to <24	7,433 (33%)
	>24	7,692 (35%)

SD, standard deviation; *IQR,* interquartile range.

Supplemental Table 3. Unadjusted and multivariable adjusted gee analyses with race/ethnicity and primary language

		Unadjusted			Model A*			Model B**			Model C***		
		estimate (min)	*P*	95% CI	estimate (min)	*P*	95% CI	estimate (min)	*P*	95% CI	estimate (min)	P	95% CI
Age (ref: 18 to 64)	65+	54	<0.001	[31, 78]	29	0.05	[0, 58]	27	0.08	[-3, 56]	25	0.10	[-5, 55]
Sex (ref: Female)	Male	5	0.68	[-19, 29]	10	0.38	[-12, 32]	10	0.38	[-12, 32]	10	0.37	[-12, 32]
Combined Race and Ethnicity (ref: Non-Hispanic White)	Hispanic	-1	0.97	[-37, 36]	-7	0.72	[-43, 30]				-19	0.35	[-60, 21]
	Black	17	0.38	[-21, 55]	14	0.45	[-22, 51]				11	0.56	[-26, 48]
	Other	11	0.64	[-35, 56]	-7	0.74	[-50, 35]				-17	0.46	[-61, 28]
Language (ref: English)	Non-English	30	0.08	[-4, 64]				15	0.37	[-17, 47]	25	0.19	[-12, 63]
Insurance (ref: Commercial)	Medicaid	96	<0.001	[60, 132]	85	<0.001	[49, 121]	81	<0.001	[45, 117]	82	<0.001	[46, 118]
	Medicare	113	<0.001	[82, 145]	67	<0.001	[32, 103]	68	<0.001	[33, 103]	68	<0.001	[32, 103]
	Other	128	0.002	[48, 208]	97	0.01	[21, 172]	92	0.02	[17, 168]	94	0.02	[18, 170]
Housing (ref: Permanent)	Non-Permanent	43	0.11	[-10, 96]	25	0.35	[-27, 76]	28	0.28	[-23, 80]	26	0.32	[-25, 78]
Special Bed Type (ref: No)	Yes	74	<0.001	[32, 116]	50	0.01	[11, 90]	50	0.01	[11, 89]	50	0.01	[11, 89]
Bed Precaution (ref: No)	Yes	47	0.52	[-95,189]	17	0.81	[-120, 155]	17	0.81	[-121, 154]	17	0.81	[-121, 154]
Bed Request Time (ref: 7a to 3p)	3p to 11p	124	<0.001	[95, 152]	232	<0.001	[204, 260]	232	<0.001	[204, 260]	232	<0.001	[204, 260]
	11p to 7a	128	<0.001	[94, 161]	177	<0.001	[145, 209]	177	<0.001	[145, 209]	177	<0.001	[145, 209]
Bed Request Day of Week (ref: Monday)	Tuesday	10	0.62	[-31, 52]	-117	<0.001	[-156, -78]	-116	<0.001	[-155, -77]	-117	<0.001	[-156, -78]
	Wednesday	-25	0.23	[-65, 15]	-160	<0.001	[-199, -122]	-160	<0.001	[-198, -121]	-160	<0.001	[-198, -122]
	Thursday	-86	<0.001	[-125, -46]	-212	<0.001	[-251, -174]	-212	<0.001	[-250, -174]	-212	<0.001	[-251, -174]
	Friday	-129	<0.001	[-172, -86]	-155	<0.001	[-197, -114]	-155	<0.001	[-196, -114]	-155	<0.001	[-197, -114]
	Saturday	64	0.008	[17, 111]	168	<0.001	[122, 214]	168	<0.001	[122, 21 4]	168	<0.001	[122, 214]
	Sunday	92	<0.001	[46, 138]	248	<0.001	[202, 294]	248	<0.001	[202, 294]	248	<0.001	[202, 293]
Active Isolation (ref: No)	Yes	132	<0.001	[101, 162]	112	<0.001	[83, 141]	112	<0.001	[83, 141]	112	<0.001	[83, 141]
Acuity per Emergency Severity Index (ref: ESI of 2)	1	133	0.12	[-36, 302]	124	0.11	[-27, 275]	123	0.11	[-28, 274]	123	0.11	[-28, 274]
	3	-144	<0.001	[-168, -119]	-131	<0.001	[-153, -108]	-130	<0.001	[-153, -108]	-131	<0.001	[-153, -108]
	4 and 5	-365	<0.001	[-445, -285]	-364	<0.001	[-441, -286]	-363	<0.001	[-441, -286]	-364	<0.001	[-441, -286]
	Unavailable	-173	0.02	[-311, -34]	-145	0.03	[-278, -11]	-146	0.03	[-279, -13]	-145	0.03	[-279, -12]
ED Census		6	<0.001	[5, 6]	1	0.003	[0, 2]	1	0.003	[0, 2]	1	0.003	[0, 2]
Boarder Census		21	<0.001	[20, 22]	25	<0.001	[23, 26]	25	<0.001	[23, 26]	25	<0.001	[23, 26]

*Model A is adjusted and includes Race/Ethnicity
**Model B is adjusted and includes Primary Language
***Model C is adjusted and includes both Race/Ethnicity and Primary Language

Supplemental Table 4. Adjusted GEE analyses among patients boarding time ≥120 minutes and patients with only a single admission order.

		**Adjusted Model of Patients Boarding 120+ minutes (n = 21,572)**			**Adjusted Model of Patients with Only a Single Admission Order Entered (n = 21,912)**		
		estimate (minutes)	p-value	95% confidence interval	estimate (minutes)	p-value	95% confidence interval
Age (ref: 18 to 64)	65+	21	0.16	[-8, 51]	27	0.08	[-3, 56]
Sex (ref: Female)	Male	18	0.12	[-5, 40]	9	0.41	[-13, 32]
Combined Race and Ethnicity (ref: Non-Hispanic White)	Hispanic	-8	0.68	[-44, 29]	-6	0.74	[-43, 30]
	Black	7	0.71	[-30, 44]	14	0.44	[-22, 51]
	Other	-10	0.66	[-52, 33]	-4	0.85	[-47, 39]
Language (ref: English)	Non-English	92	<0.001	[56, 128]	87	<0.001	[50, 123]
Insurance (ref: Commercial)	Medicaid	72	<0.001	[36, 108]	71	<0.001	[35, 107]
	Medicare	117	0.003	[40, 193]	91	0.02	[15, 167]
	Other	23	0.39	[-29, 74]	25	0.35	[-27, 76]
Housing (ref: Permanent)	Non-Permanent	54	0.008	[14, 93]	51	0.01	[11, 91]
Special Bed Type (ref: No)	Yes	35	0.62	[-102, 172]	27	0.70	[-112, 166]
Bed Precaution (ref: No)	Yes	241	<0.001	[213, 270]	230	<0.001	[201, 258]
Bed Request Time (ref: 7a to 3p)	3p to 11p	163	<0.001	[131, 195]	175	<0.001	[143, 208]
	11p to 7a	-101	<0.001	[-140, -62]	-120	<0.001	[-159, -81]
Bed Request Day of Week (ref: Monday)	Tuesday	-165	<0.001	[-204, -127]	-161	<0.001	[-200, -122]
	Wednesday	-212	<0.001	[-250, -173]	-209	<0.001	[-248, -171]
	Thursday	-145	<0.001	[-187, -103]	-154	<0.001	[-196, -112]
	Friday	173	<0.001	[126, 220]	166	<0.001	[119, 213]
	Saturday	259	<0.001	[213, 306]	243	<0.001	[197, 289]
	Sunday	125	<0.001	[96, 154]	112	<0.001	[83, 141]
Active Isolation (ref: No)	Yes	119	0.12	[-32, 271]	124	0.12	[-31, 278]
Acuity per Emergency Severity Index (ref: ESI of 2)	1	-132	<0.001	[-154, -109]	-134	<0.001	[-157, -111]
	3	-366	<0.001	[-444, -288]	-369	<0.001	[-448, -291]
	4 and 5	-107	0.12	[-244, 29]	-141	0.04	[-276, -6]
	Unavailable	21	0.16	[-8, 51]	27	0.08	[-3, 56]
	ED Census	1	0.001	[0, 2]	1	0.003	[0, 2]
	Boarder Census	23	<0.001	[22, 25]	25	<0.001	[23, 26]

Supplemental Table 5. Insurance carrier stratified by age

Age in years	Commercial	Medicaid	Medicare	Other
18-64	2,987 (82%)	4,508 (82%)	2,413 (19%)	417 (64%)
65+	666 (18%)	968 (18%)	10,099 (81%)	233 (36%)
